# The Impact of Long-Term Antibiotic Therapy of Cutaneous Adverse Reactions to EGFR Inhibitors in Colorectal Cancer Patients

**DOI:** 10.3390/jcm10153219

**Published:** 2021-07-21

**Authors:** Mara Mădălina Mihai, Ana Ion, Călin Giurcăneanu, Cornelia Nițipir, Ana-Maria Popa, Mariana-Carmen Chifiriuc, Mircea Ioan Popa, Jan Říčař, Liliana Gabriela Popa, Ionela Sârbu, Veronica Lazăr

**Affiliations:** 1 Department of Oncologic Dermatology, ‘Elias’ Emergency University Hospital, ‘Carol Davila’ University of Medicine and Pharmacy, 020021 Bucharest, Romania; calin.giurcaneanu@gmail.com (C.G.); liliana.popa@umfcd.ro (L.G.P.); 2 Department of Dermatology, ‘Elias’ Emergency University Hospital, 011461 Bucharest, Romania; 3 Department of Microbiology, Faculty of Biology, ICUB—Research Institute of the University of Bucharest, 050657 Bucharest, Romania; carmen.chifiriuc@bio.unibuc.ro (M.-C.C.); veronica.lazar2009@gmail.com (V.L.); 4 Department of Oncology, ‘Elias’ Emergency University Hospital, ‘Carol Davila’ University of Medicine and Pharmacy, 020021 Bucharest, Romania; cornelia.nitipir@umfcd.ro (C.N.); ana-maria.popa@umfcd.ro (A.-M.P.); 5 Department of Microbiology, Faculty of Medicine, ‘Carol Davila’ University of Medicine and Pharmacy, 020021 Bucharest, Romania; mircea.ioan.popa@umfcd.ro; 6 Department of Dermatology and Venereology, Charles University, Medical School and Teaching Hospital Pilsen, 30599 Pilsen, Czech Republic; ricarj@fnplzen.cz; 7 Department of Genetics, Faculty of Biology, ICUB—Research Institute of the University of Bucharest, 050657 Bucharest, Romania; ionela24avram@yahoo.com

**Keywords:** gut microbiome dysbiosis, *Fusobacterium nucleatum*, metastatic colorectal cancer, EGFR inhibitors, tetracyclines, papulo-pustular rash, acneiform rash, long-term antibiotic therapy

## Abstract

Colorectal cancer (CRC) is an important public health issue, in terms of incidence and mortality, with approximately 1.8 million new cases reported worldwide in 2018. Advancements in understanding pathophysiological key steps in CRC tumorigenesis have led to the development of new targeted therapies such as those based on epidermal growth factor receptor inhibitors (EGFR inhibitors). The cutaneous adverse reactions induced by EGFR inhibitors, particularly papulopustular rash, often require long-term antibiotic treatment with tetracycline agents (mostly minocycline and doxycycline). However, this raises several issues of concern: possible occurrence of gut dysbiosis in already vulnerable CRC patients, selection of highly antibiotic resistant and/or virulent clones, development of adverse reactions related to tetracyclines, interference of antibiotics with the response to oncologic therapy, with a negative impact on disease prognosis etc. In the context of scarce information regarding these issues and controversial opinions regarding the role of tetracyclines in patients under EGFR inhibitors, our aim was to perform a thorough literature review and discuss the main challenges raised by long-term use of tetracyclines in advanced CRC patients receiving this targeted therapy.

## 1. Introduction

Colorectal cancer (CRC) is an important public health issue, ranking as third when referring to incidence and second in terms of mortality, with approximately 1.8 million new CRC cases arising in 2018 [1]. Advancement in understanding pathophysiological key steps in CRC tumorigenesis have led to the development of new therapeutic options, such as targeted therapy, that have increased the overall survival and progression-free survival [2]. Epidermal growth factor receptor inhibitors (EGFR inhibitors) represent a targeted therapy used for patients with advanced CRC and encompass two principal categories: anti-EGFR monoclonal antibodies and tyrosine kinase inhibitors [3].

The use of EGFR inhibitors is associated with a broad spectrum of adverse reactions, including cutaneous manifestations, occurring in up to 100% of patients, the most frequent being the papulopustular rash [4,5,6,7,8,9,10,11,12]. This eruption appears one or two weeks after the initiation of targeted-therapy for metastatic CRC patients, occurring as multiple pruritic or tender papules and sterile pustules on an erythematous background located on the scalp, face, upper chest and back [13]. This particular type of cutaneous adverse reaction often requires a six to eight weeks of antibiotic treatment with tetracycline agents (mostly minocycline and doxycycline) or even more in severe cases [14,15,16,17]. On the other side, tetracyclines, apart from their antibacterial and anti-inflammatory action [18,19,20], may have other beneficial effects such as angiogenesis inhibition [18,19] and anti-proliferative effects on malignant cells [21].

However, despite recommendations of recent guidelines [16,17], the use of long-term antibiotherapy raises several issues of concern: the gut dysbiosis in already vulnerable patients with CRC, the development of adverse reactions related to tetracyclines, the selective pressure of antibiotic that may increase resistant bacteria ‘fitness’, leading to emergence of highly resistant and virulent clones, the negative impact of antibiotics on the response to oncologic therapy and disease prognosis, and others. There is scarce information in the scientific literature on these issues and there are controversies regarding the role of tetracyclines in patients under EGFR-inhibitors therapy. Therefore clinicians (oncologists, dermatologists, infectious diseases specialists and others) may find difficulties in the management of cutaneous reactions of these patients, addressing individual needs and concerns.

In this article we aim to perform a thorough literature review and discuss the main issues of concern raised by long-term use of tetracycline antibiotherapy in advanced CRC patients under EGFR inhibitors.

## 2. Antibiotic Treatment for Cutaneous Adverse Reactions of EGFR Inhibitors in CRC Patients

### 2.1. EGFR Inhibitors–Targeted-Therapy for Metastatic CRC Patients

EGFR plays an important role in the pathophysiology of carcinogenesis, leading to downstream activation of the RAS/RAF signaling pathway which regulates cell proliferation, survival and invasion, angiogenesis and immune evasion, ultimately favoring tumor progression [22,23]. EGFR is involved in the pathogenesis and progression of different cancers such as non-small cell lung, breast, ovarian and CRC [24].

In recent years, the approval of new, targeted therapies, has led to a significant improvement of outcomes in patients with metastatic colorectal cancer (mCRC), increasing the overall survival (OS) when associated with classic chemotherapy regimens [25].

EGFR inhibitors used as part of the therapeutic strategy in the management of metastatic CRC prevent the effects of EGFR receptor activation: cell proliferation, differentiation and angiogenesis and induce the cellular apoptosis [26,27].

There are two categories of EGFR-inhibitors: anti-EGFR monoclonal antibodies (e.g., cetuximab, panitumumab, matuzumab) which are specific for the extracellular tyrosine kinase domain of the EGF receptor and tyrosine kinase inhibitors (e.g., erlotinib, lapatinib, agatinib, gefitinib) which are directed against the intracellular tyrosine kinase domain of the EGFR receptor [28,29]. Anti-EGFR antibodies such as cetuximab or panitumumab act by blocking ligand binding, this way inhibiting the RAS-RAF-MEK-ERK signaling pathway [30]. Cetuximab is a chimeric IgG1 antibody that prevents the EGF receptor from adopting an extended conformation, thereby inhibiting EGFR activation [31]. Panitumumab is a fully human IgG2 monoclonal antibody that acts similarly to cetuximab but does not mediate antibody dependent cellular cytotoxicity [32]. The effectiveness of both drugs for patients with wild-type RAS mCRC has been firmly established by several clinical trials when used in combination with fluorouracil plus oxaliplatin (FOLFOX) or fluorouracil plus irinotecan (FOLFIRI) [33,34]. Furthermore, it has been demonstrated that the association of cetuximab to FOLFIRI as first-line therapy in patients with KRAS wild-type mCRC improves overall survival [35]. Additionally, the PRIME study showed that the association between panitumumab and FOLFOX4 as first-line therapy is not only well tolerated, but also it had a significant impact on progression free survival (PFS) in patients with wild-type KRAS colorectal tumors [33].

### 2.2. Dermatological Adverse Reactions of EGFR Inhibitors

EGFR is highly expressed in the normal skin and plays an important part in its development and physiology. EGFR can be found in the basal and suprabasal keratinocytes, sebaceous epithelium, dendritic cells, outer root sheath of hair follicles [36,37]. Consequently, cutaneous complications are the most frequent adverse side effects of EGFR inhibitors, occurring in up to 100% of patients treated with cetuximab and panitumumab therapy [4,5,6]. Such cutaneous clinical findings are papulopustular eruptions, photosensitivity, hair and nail changes, mucosal changes, xerosis, eczema and fissures, hyperpigmentation, telangiectasia and pruritus [7,8,9,10,11,12,38,39,40,41]. Rarely, serious adverse reactions such as Stevens Johnson Syndrome (SJS), toxic epidermal necrolysis (TEN) and acute generalized exanthematous pustulosis (AGEP) have been described in EGFR inhibitors treated patients [7,8,9,10,11,12,38,39,40,41].

However, the most frequent cutaneous adverse reaction is the papulopustular rash, occurring in up to two-third of patients treated with small molecule inhibitors and up to 90% of patients treated with monoclonal antibodies [7,8,9,10,11,12,42,43].

Skin toxicity of novel therapeutic agents significantly impairs the quality of life (QOL) of cancer patients compared with non-targeted therapy, the EGFR inhibitor rash and pruritus being associated with the highest negative impact [44]. A clinical trial from 2016 shows that papulopustular eruption, along with pruritus and xerosis represent major EGFR-associated cutaneous adverse effects, which have a significant negative effect on health-related quality of life [45]. Therefore, such manifestations might warrant more attention in clinical practice in order to adequately manage such complex cases [45].

### 2.3. Tetracyclines as Elected Therapy in the Management of Papulo-Pustular Rash to EGFR-Inhibitors

Current approach to anti-EGFR therapy-related papulo-pustular reactions can be either reactive or pre-emptive [46]. Reactive treatment is administered only when an adverse skin reaction appears [47]. The pre-emptive approach is a prophylactic one, used to prevent the appearance of cutaneous manifestations under EGFR–inhibitors and consists of oral antibiotics, either alone or in combination with topical corticosteroids or non-pharmacological prophylactic measures (such as the use of moisturizers and sunscreens) [17]. The most frequently recommended antibiotics for reactive or pre-emptive treatment are tetracyclines. Aside from their already well-known antibacterial activity, tetracyclines have several other roles such as anti-inflammatory properties by the inhibition of metaloproteinases and pro-inflammatory cytokines and by the inhibition of lymphocyte proliferation and neutrophil migration; experimental data also suggests that doxycycline and minocycline may inhibit angiogenesis [18,19,20].

A study on human CRC cell lines demonstrated that doxycycline, apart from its antimicrobial activity, exerted an antiproliferative and anti-invasion effect especially when combined with a cyclooxigenase 2 (COX-2) inhibitor [21]. Sagar J et al. showed that doxycycline induced apoptosis in CRC cells when combined with platinum agents, perhaps via activation of caspase 3, without any additive or synergic effects [48]. A review by Ali I et al. suggested that doxycycline has an anti-matrix metalloproteinases (MMPs) effect, cytotoxic effect on neoplastic cells and the ability to sensitize some cancer cells for radiation therapy and thereby, it could act as a potential anti-cancer agent [49].

The STEPP trial was among the first to demonstrate the benefits of pre-emptive treatment in patients with metastatic colonic cancer under EGFR-inhibitors. The pre-emptive treatment consisted in the administration of oral doxycycline 100 mg twice per day, moisturizers, sunscreen, topical corticosteroids (hydrocortisone cream 1%) one day before the initiation of panitumumab up until six weeks, thereby significantly reducing the incidence of protocol-specified grade ≥2 skin toxicities vs. reactive treatment (29% in the pre-emptive arm vs. 62% in the reactive arm) [50].

Several other randomized controlled trials have also demonstrated the efficacy of systemic antibiotics for the prophylaxis of EGFR- inhibitors associated skin toxicity in metastatic CRC patients, with tetracyclines (particularly minocycline and doxycycline) being the preferred antibiotic class. In 2015, Yamada M et al. showed that pre-emptive antibiotic therapy with minocycline (100 mg once a day) and proper skin care reduced the incidence of grade ≥2 acneiform rash in pre-emptive group compared to the reactive group (44% vs. 84.6%) [51]. A placebo-controlled study from 2008 used tetracycline (500 mg, twice per day for four weeks) as the main antibiotic agent in the pre-emptive approach for cancer patients under EGFR inhibitors and although it did not prevent the occurrence of skin rash, it diminished its severity together with other symptoms, such as skin burning or skin irritation improved [52]. The J-STEPP study demonstrated that pre-emptive treatment (skin care along with systemic minocycline -100 mg once a day from the day before the initiation of Panitumumab-chemotherapy regimen to the following six weeks) resulted in a lower incidence of grade ≥2 skin toxicities compared with reactive treatment of cutaneous manifestations [53].

On the other side, there are some reports that showed opposite results. A randomized, double-blind, placebo-controlled study by Jatoi A et al. from 2011 found that tetracyclines did not diminish the incidence or severity of EGFR inhibitors induced rash: from the 65 patients enrolled, 27 treated with tetracycline (82%) and 24 placebo-exposed patients (75%) developed a rash during the first four weeks of the EGFR treatment. The rash was grade 2+ and was also observed through week five to week eight of EGFR inhibitors therapy [54].

There are few official guidelines or proposed guidelines on the use of antibiotics in EGFR inhibitors induced papulo-pustular rash [17]. An official guideline from Albertha Health Services mentions that at the beginning of the EGFR therapy, antibiotics may be used in order to reduce the incidence and the severity of the acneiform rash, tetracyclines such as minocycline (100–200 mg daily) or doxycycline (100–200 mg daily) being the recommended class.16 For patients with either allergies or intolerance to minocycline or doxycycline, erythromycin 500 mg twice a day or trimethoprim 160 mg/sulfamethoxazole 800 mg twice a day may also be used [16]. Concerning the management of the papulopustular lesions, systemic antibiotic therapy with minocycline or doxycycline 100 mg twice daily for four weeks along with a proper topical therapy with hydrocortisone 1% cream and clindamycin 2% is recommended for grade 2 rashes [16]. For rashes grade ≥3, in addition to the above-mentioned therapies, oral prednisone up to 0.5 mg/kg daily for seven to 14 days may be employed and also dose reduction of the EGFR inhibitor may be required [16]. If after four weeks the rash still persists or worsens, isotretinoin 20–30 mg daily or acitretin 25 mg daily may be considered [16]. If the acneiform rash does not improve or worsens after EGFR inhibitor dose reduction and the various therapeutic options mentioned anteriorly, complete discontinuation of EGFR inhibitors may be mandatory [16]. Prophylactic photoprotective measures and avoidance of skin irritants are important recommendations together with topical therapy, including corticosteroids and metronidazole creams, ointments or other compounded formulas [17].

A review from 2016 by Hofheinz R-D et al. suggests that prophylactic care from the first day of EGFR inhibitor therapy may reduce the severity of the cutaneous adverse reactions up to twofold or greater. Consequently, sunlight protection, a skincare regimen based on gentle cleansing and hydrophilic cream, along with oral prophylactic antibiotic therapy with minocycline 100 mg daily or doxycycline 100–200 mg daily for ≥8 weeks are recommended [17]. This review highlights that antibiotic therapy should be started on the first day of EGFR inhibitor treatment [17].

In a clinical trial from 2008, Racca P et al. found that cooperation between oncologists and dermatology specialists is very important in order to correctly identify and treat EGFR cutaneous side effects [55]. It should be emphasized that antibiotics are recommended to be employed only when referring to papulopustular eruption, rather than other cutaneous adverse reactions [16].

However, the side effects of antibiotics must also be taken into consideration. Common adverse reactions to doxycycline are heartburn and the gastrointestinal ones, such as gastritis, nausea, vomiting, diarrhea [56]. Doxycycline is considered to be involved in the majority of the reported drug-induced esophageal ulcerations, up to 70% [57]. Cutaneous adverse reactions are the second most common side effects of doxycycline and include pruritus, rashes and photosensitivity [58]. It has been shown that photosensitivity is dose dependent, occurring in 42% of the patients taking 200 mg doxycycline daily [59]. Less common side effects of doxycycline include benign intracranial hypertension and hematologic disorders, such as hemolytic anemia, thrombocytopenia and neutropenia [60,61]. Minocycline is associated with the following adverse reactions: hyperpigmentation in skin, nail beds, teeth, mucous membranes; sensitization, which may occur weeks after the beginning of the antibiotic therapy; autoimmune diseases such as drug-related lupus [62]. The skin pigmentation seen during long-term administration of minocycline is not usual with doxycycline [60].

A randomized phase II trial from 2017 by Kripp M et al. was the first to compare the administration of oral doxycycline vs. local erythromycin as pre-emptive therapeutic approach of panitumumab-associated skin eruptions in patients with mCRC [15]. Results showed that significantly more patients in the erythromycin group developed moderate to severe skin eruptions at earlier time points, even though overall quality of life was similar between the two groups [15].

## 3. The Impact of Long-Term Antibiotherapy on the Intestinal Microbiome in CRC Patients

### 3.1. Intestinal Microbiome and CRC

The intestinal microbiome refers to a complex and dynamic microenvironment composed of bacteria, archeae, fungi and viruses inhabiting the human intestinal tract [63]. According to recent studies based on shot-gun sequencing analysis, each individual harbors unique profiles of gut microbes. It seems that we can have between 58 to 346 different bacterial species in the human gut, thus being very difficult to understand the role that each strain plays in intestinal homeostasis, but even so human gut dysbiosis has been associated with several diseases including cancer [64].

The etiopathology of CRC is still not completely elucidated but, along with genetic and lifestyle factors, dysbiosis also plays a key part in the development of CRC [65]. Furthermore, environmental factors that are believed to increase the risk of CRC may, in fact, produce changes in the composition of intestinal microbiome [66]. In vitro and in vivo studies highlighted that some microbial strains from human gut may contribute to the development of CRC by releasing metabolites that affect host cells’ functions [67,68]. An example is *Enterococcus faecalis* and *Bacteroides fragilis* which release reactive oxygen species and enterotoxins that lead to oxidative DNA damage, destruction of the epithelial barrier and activation of inflammatory cascades [69,70]. An experimental study from 2016 showed that *Fusobacterium nucleatum* increased proliferation of CRC cells, leading to a faster growth of a larger tumor than controls via TLR-4 (Toll-like receptor 4) signaling to NFkB (nuclear factor kappa-light-chain-enhancer of activated B cells), upregulating expression of microRNA-21 [71].

**Figure 1:**
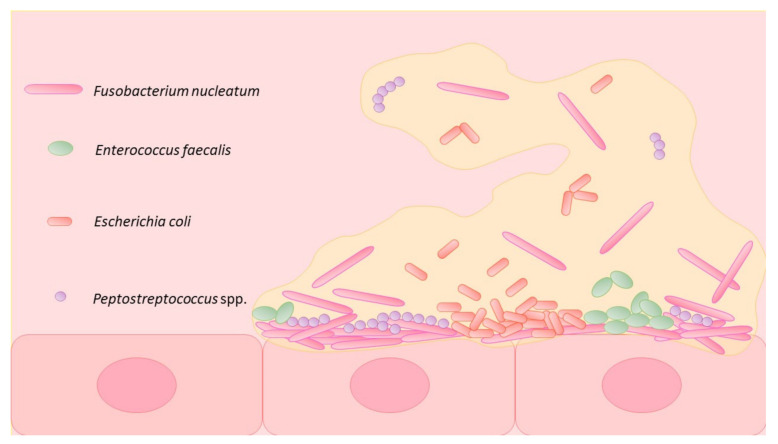
Gut microbiome diversity in colorectal cancer patients.

High-throughput DNA sequencing analysis performed on stool samples from CRC patients revealed that the gut microbiome is significantly different from that of healthy adults [72] and studies on tumor-bearing animal models shows that the gut microbiota in CRC is essential for the anti-tumor effect of PD-1 antibody therapy [73] (Figure 1).

An increased abundance of *Fusobacterium nucleatum*, *Bacteroides fragilis*, *Enterococcus faecalis*, *Escherichia coli*, *Peptostreptococcus stomatitis* and *Parvimonas micra* was observed in CRC patients, while other species from Bifidobacterium, Clostridiales, Faecalibacterium or Blautia genera are absent or in lower numbers [74,75]. Given that emerging data highlight the association between intestinal dysbiosis and colorectal tumorigenesis, evaluation of microbial markers may be considered in early CRC screenings. Metagenome-wide association studies on fecal samples have shown that *Fusobacterium nucleatum* is the main bacterial species present in high amounts in both CRC and adenomas microenvironments (tumor tissues, as well as in stool specimens of CRC patients) and it has been demonstrated that it plays a crucial part in colorectal tumorigenesis as well as in chemoresistance and prognosis in CRC patients [71,76,77,78,79,80]. Consequently, this bacterium species may be the subject of further study concerning biomarkers for early non-invasive CRC diagnosis. Therefore, *Fusobacterium nucleatum* specific antibiotherapy may have beneficial effects on the prognosis of CRC patients.

In an experimental study, Bullman S et al. found that *Fusobacterium nucleatum* survived in CRC patient derived xenografts (PDXs) during multiple generations and treatment with a *Fusobacterium*-bearing PDX model with metronidazole decreased *Fusobacterium* load and malignant cell proliferation; these findings provide a strong basis for future research regarding the development of *Fusobacterium* specific therapies [81].

Another experimental study demonstrated not only that *Fusobacterium nucleatum* favored resistance to chemotherapy (mainly 5-fluorouracil, capecitabine and oxaliplatin) in advanced CRC patients, but also that *Fusobacterium nucleatum* was found in high amounts in cancer tissues of patients with post-chemotherapy recurrence and was associated with a poor prognosis [76]. Chen Y et al. showed the impact of *Fusobacterium nucleatum* on the prognosis of stage III or high-risk stage II CRC patients: the difference between Fusobacterium-high group and Fusobacterium-low/negative group regarding OS was significant, a high amount of *Fusobacterium nucleatum* being associated with a shorter surviving time [82].

A study from 2020 by Liu Y et al. assessed the relationship between chemotherapeutic resistance in esophageal squamous cell carcinoma (ESCC) and *Fusobacterium nucleatum*. Results showed that ESCC patients which had *Fusobacterium nucleatum* infection presented lesser therapeutic response to chemotherapy, mostly to 5-fluorouracil, cisplatin and docetaxel, due to the modulation of autophagosome formation induced by *Fusobacterium nucleatum* [83].

Concerning EGFR inhibitors, there is not sufficient data to establish whether *Fusobacterium nucleatum* may influence directly the efficacy of these targeted therapies or the prognosis in mCRC.

Considering that *Fusobacterium nucleatum* is considered to play an essential part in the pathophysiology of CRC, antibiotic therapy with agents covering this bacterial genus, may be beneficial for the patients. There are no official guidelines on the treatment of *Fusobacterium nucleatum* infections. According to the Sanford Guide to antimicrobial therapy 2020, the elected therapy for *Fusobacterium necrophorum* infections includes the following antibiotics: chloramphenicol, clindamycin, tetracyclines and metronidazole [84]. Moreover, there are guidelines for the treatment of Lemierre ‘s syndrome also called jugular vein suppurative phlebitis, an acute infectious disease caused more frequently by *Fusobacterium necrophorum*, but also by *Fusobacterium nucleatum* or other bacterial species [85]. According to the Sanford Guide to antimicrobial therapy 2020, the syndrome should be treated with piperacillin-tazobactam 4.5 gm at every eight hours or IMP 500 mg intravenously every six hours or metronidazole 500 mg orally or intravenously every eight hours and ceftriaxone 2 g intravenously once daily. The suggested alternative is clindamycin 600–900 mg intravenously every eight hours [84]. It is recommended to avoid macrolides, since *Fusobacterium* is resistant to this class of antibiotics [84]. While these antibiotics with bactericidal properties and intravenous administration are useful for acute infections, they are not feasible in CRC patients. Bacteriostatic agents with oral administration, such as tetracyclines proved to be more useful in the management of chronic infections with *Fusobacterium* spp. and were also recommended by the Sanford Guide to antimicrobial therapy 2020 [84]. The anti-anaerobic and particularly anti-*Fusobacterium* activity of doxycycline could bring a potential benefit for patients with mCRC under EGFR inhibitors receiving this antibiotic agent for skin rashes.

### 3.2. Gut Microbiome Manipulation in mCRC Patients

Manipulation of gut microbiome in mCRC patients may represent an effective solution for the pre-existing dysbiosis. Along with diet, the main therapeutic methods which modify the gut microenvironment are fecal microbiota transplantation (FMT) and the use of probiotics, prebiotics, postbiotics and synbiotics [86,87,88,89,90,91].

Diet plays an essential part in microbiome modulation and, consequently, in the therapeutic approach of CRC patients. In a study from 2017, Mehta RS et al. showed that a diet rich in whole grains and dietary fiber was associated with a lower risk for *Fusobacterium nucleatum*-positive CRC [92]. This finding suggests that gut microbiota may mediate the correlation between diet and CRC [92]. There are no clearly established guidelines on the right type of diet that may have a significant impact on cancer incidence. Nevertheless, it seems that a reduced caloric intake increases the efficacy of cancer treatment [93]. This is the case for every-other-day fasting, which has been shown to elevate gut acetate and lactate, increase the level of *Firmicutes* and decrease *Bacteroidetes, Tenericutes* and *Actinobacteria* [93]. In an experimental study from 2015, Hao GW et al. found that an unrestricted ketogenic diet led to a delay in tumor growth in a mouse xenograft model [94]. Ketogenic diet (KD) may be a reasonable dietary approach in the management of mCRC since it mimics the metabolic state of fasting by increasing the levels of beta-hydroxybutyrate and acetoacetate [94].

The correlation between diet and its impact on gut microbiome, dysbiosis, cancer prevention and cancer treatment is a challenging field of research, nevertheless, experimental and clinical studies may, in a near future, unveil novel findings with therapeutic applicability on this particular matter.

In the last years, attention has been brought on the potential prophylactic role of probiotics, since dysbiosis is believed to play an important part in colorectal carcinogenesis [95,96,97].

Probiotic bacteria may be defined as: ‘live microorganisms which when administered in adequate amounts confer a health benefit on the host’ [98]. It has been demonstrated that probiotics may inhibit the tyrosine kinase signaling pathway and EGFR activation. A laboratory study by Ma LE et al. showed that *Bacillus polyfermenticus* exerted an antitumoral effect both in vitro and in vivo by reducing the activity of ErbB2 and ErbB3, as well as their signaling molecules E2F-1 and cyclin D1 [99]. Several other mechanisms are believed to be responsible of the potential preventive role of probiotics in CRC: competition with pathogenic microbiota, regulation of cell differentiation, apoptosis and inactivation of carcinogenic compounds [98]. In a clinical trial from 2015, Kotzampassi K et al. showed that the use of probiotics reduced the rate of all postoperative major complications (probiotics 28.6% vs. placebo 48.8%) in CRC patients [100]. A randomized, double-blind clinical trial showed that synbiotics markedly reduced the postoperative infectious complications in CRC patients [101]. Nevertheless, conclusive clinical evidence suggesting the preventive role of probiotics in CRC is not currently available. Moreover, incontestable evidence regarding the safety of probiotics, prebiotics and synbiotics in mCRC patients is not available at the moment.

Regarding FMT, even though this method has not been extensively studied in CRC, a publication showed beneficial effects of this therapeutic method in the treatment of inflammatory bowel diseases, intractable functional constipation and hematologic malignancies [102]. Consequently, FMT may represent a potential answer for the dysbiosis found in CRC patients or, particularly, in those treated with long-term antibiotic therapy for acneiform rash induced by EGFR inhibitors, with conclusive evidence needed to further demonstrate this hypothesis.

Until now the issue of gut microbiome manipulation has been raised mainly in the prevention of CRC. However, the multiple options (diet, fecal microbiota transplantation (FMT), probiotics, prebiotics, postbiotics, synbiotics) may be considered in patients already diagnosed with CRC, treated with EGFR inhibitors and with antibiotic therapy for cutaneous adverse reactions. Whether these interventions may have an impact on the therapeutic response or on the long-term survival remains to be established in future studies.

### 3.3. Impact of Long-Term Tetracyclines on Gut Microbiome in Metastatic CRC Patients

As it was mentioned above, tetracyclines are the preferred agents for treating skin rashes induced by EGFR inhibitors and might be of a potential benefit for patients with mCRC. However, systemic therapy could alter the gut microbiome.

In a clinical study from 2014, Angelakis E et al. included 82 patients diagnosed with Q fever endocarditis and demonstrated that prolonged administration of doxycycline (100 mg twice a day, for at least 12 months) and hydroxychloroquine was associated with an important decrease in *Bacteroidetes* spp., *Firmicutes* spp. and *Lactobacillus* spp., as well as the overall bacterial intestinal content and diversity, as shown by real-time PCR assay from purified DNA of fecal samples [103].

A systematic review from September 2020 by Elvers KT et al. mentioned the changes induced by doxycycline on gut microbiome: at suboptimal dosage (20 mg or 40 mg daily for 9 months and, respectively, 16 weeks), it reduces enterococci and *Escherichia coli* and at normal dosage (100–150 mg daily for 7–10 days) it eliminates *Fusobacterium* species and markedly affects the *Bifidobacteria* populations diversity [104]. Moreover, there was observed an increase of *Bifidobacteria* tetracycline resistant strains in the group of patients treated with doxycycline [104].

A case-control study from 2020 by Thompson KG et al. on eight acne patients investigated the modifications in skin and gut microbiome after receiving oral minocycline. Results demonstrated that there was a significant depletion in *Bifidobacterium breve* (*p* = 0.042), *Bifidobacterium pseudolongum* (*p* = 0.010) and *Lactobacillus salivarius* (*p* = 0.001) [105].

A systematic review from 2019 by Zimmermann P and Curtis N describes clearly the impact of antibiotic agents on the composition of intestinal microbiota [106]. Amoxicillin, cephalosporins, clindamycin, quinolones and macrolides increased the amount of *Enterobacter* spp., *Klebsiella* spp., *Citrobacter* spp. and decreased the abundance of *Escherichia coli* [106]. In contrast to other penicillins, amoxicillin/clavulanate increased the abundance of *Escherichia coli* [106]. Doxycycline decreased the amount of *Enterococcus* spp. [106].

A randomized placebo-controlled clinical trial by Zaura et al. from 2015 which included 66 patients receiving either ciprofloxacin, minocycline, clindamycin, amoxicillin or a placebo showed that after one year no significant modifications in the intestinal microbiome were detected [107]. It seems that alterations in gut microbiome are seen for a short time, up to one month, except for clindamycin, where effects can be seen four months after the antibiotic therapy [107].

The long-term antibiotic therapy may alter not only the abundance of some species, but also the bacterial fitness, defined as the ability of bacteria to adjust their metabolism to adequately suit environmental conditions, thereby being able to survive and grow; this concept is considered a major physiological determinant [108,109,110]. Under certain conditions, including antibiotic therapy, bacteria with low fitness could be favorized, having significantly high and fluctuating gene expression changes that exceed bacteria with high level fitness [111,112,113]. Regarding possible modifications of gene expression of the gut bacteria in patients with CRC under antibiotic therapy, further research is needed to demonstrate these potential changes and also their impact on microbial virulence, antibiotic resistance and overall outcome of the patients.

### 3.4. Impact of Long-Term Antibiotic Therapy on CRC Development

Studies suggest that frequent antibiotic exposure may actually lead to the development of CRC. Therefore, the influence of long-term antibiotic therapy on the intestinal microbiome of CRP patients should not be neglected, since dysbiosis is already present in this type of cancer.

A Finnish cohort study from 2008 by Kilkkinen A et al. (*n* = 3,112,624) found a relative risk of 1.15 for developing CRC and 1.37 for developing any type of cancer for patients who received more than 6 antibiotic prescriptions compared to those who received 0–1 prescription in a three-year period prior to cancer diagnosis [114]. A nested case-control study from 2015 on 20,017 subjects found a significant association between the number of antibiotic prescriptions and the risk of CRC for both anti-aerobic and anti-anaerobic agents [115]. When referring to classes of antibiotics, it seemed that penicillins and quinolones had a high risk of promoting CRC development, while tetracyclines showed a low risk [115].

A matched case-control study from 2019 by Zhang J et al. assessed the risk of CRC due to oral antibiotic use [116]. Results showed that antibiotics increased the colon cancer risk in a dose-dependent manner, but decreased the appearance of CRC cancer, which was also available for tetracyclines [116].

### 3.5. Long-Term Antibiotherapy May Impact the Response to Oncologic Therapy in Metastatic CRC Patients

Long-term antibiotherapy associated modifications in the gut microbiome of CRC patients might influence the therapeutic response to EGFR inhibitors or other therapies frequently used as second line or third line treatments, such as immunotherapy.

As mentioned above, bevacizumab in combination with chemotherapy represents a first-line therapeutic alternative for patients with RAS metastatic CRC. A retrospective, single-center cohort study from 2019 by Lu L et al. assessed the association between antibiotic therapy (tetracyclines included) and mortality in mCRC patients treated with bevacizumab-containing therapy: results showed an inverse association between antibiotic exposure and mortality risk [117]. This finding is in accordance with some preclinical studies [76,81].

A cohort study from 2020 by Nenclares P et al. performed on patients with locally advanced head and neck cancer (LAHNC) which received curative chemotherapy and radiation therapy (from which one subject received cetuximab and radiation therapy) evaluated the impact of antibiotic exposure on clinical outcomes [118]. Results showed that patients who received antibiotic therapy one week before and two weeks after the treatment had a significantly reduced PFS, OS and disease-specific survival (DSS) and, moreover, antibiotic exposure was associated with local and regional tumor relapse [118]. This data may represent a starting point for future research concerning the association between antibiotic therapy, the possibility of tumor relapse and outcomes in mCRC patients treated with cetuximab or other EGFR inhibitors who are also receiving antibiotics.

At present, experimental studies pointing in the direction of immunotherapy, particularly, for immune checkpoint inhibitors (ICIs). ICIs novel therapeutic agents which target T-cell checkpoint pathways, such as CTLA-4 (cytotoxic T-lymphocyte antigen-4) and PD-1 (programmed death-1) [119]. When referring to CRC, the first FDA-approved anti PD-1 agents for the therapeutic management of deficient mismatch repair (dMMR) mCRC were nivolumab and pembrolizumab [120]. In April 2020, Xu X et al. performed a study on mice in which the effect of gut microbiome on the efficacy of PD-1 antibody immunotherapy in MSS-type mouse colon carcinoma cell lines was assessed [73]. The anti-tumor effect of PD-1 antibody therapy was maintained for the mice group treated with sterile water, medium for the mice group treated with vancomycin and poor in the mice group treated with colistin [73]. The mice group treated with broad-spectrum antibiotics did not respond at all to PD-1 antibody therapy [73]. Results indicated that a well-balanced gut microbiome is crucial for obtaining optimal anti-tumor effects of immunotherapy [73]. Consequently, it may be hypothesized that patients with long-term antibiotic therapy for EGFR inhibitors papulo-pustular rash who undergo a therapeutic switch towards immunotherapy, may have an inferior response compared to antibiotic naive patients.

## 4. Alternatives to Antibiotic Treatment

With respect to potential alternatives for long-term antibiotic therapy for cutaneous adverse reactions of mCRC patients under EGFR inhibitors, topical therapy may be a feasible option. However, this could lead to an increase in the incidence of severe acneiform eruptions (≥grade 3) and to the discontinuation, interruption or dose reduction of EGFR inhibitors.

In a review from 2016, Kozuki T et al. reported that corticosteroid therapy may represent another therapeutic option for the management of skin toxicity in patients with metastatic CRC under EGFR inhibitors [46]. Generally, topical steroid ointments or creams may be prescribed by clinicians for acneiform eruptions, while systemic dexamethasone or prednisolone are usually employed when patients experience a severe, grade 3 or 4, skin rash [46]. In 2019 Annuziata MC et al. showed favorable results in five patients with grade 1 an acneiform rash, treated with fusidic acid plus betamethasone in a lipid-enriched topical formulation [47].

Regarding topical retinoids, a randomized double-blind clinical trial from 2007 by Scope A et al. whose purpose was to evaluate the efficacy of oral minocycline, topical tazarotene or both to prevent or reduce the cetuximab-associated acne-like eruption in patients with mCRC when administered starting on the first day of the cetuximab therapy, for the following eight weeks showed topical tazarotene did not have any clinical benefit [121].

Topical calcineurin inhibitors have also been proposed as an alternative topical therapy but with limited efficacy. In 2010, a case series study by Nikolaou V et al. performed on 20 cancer patients under EGFR inhibitors evaluated the efficacy and safety of pimecrolimus 1% cream applied twice daily in the management of papulopustular eruptions caused by these novel therapeutic agents [122]. Results showed that four out of nine patients with grade 1 eruption experienced complete resolution after two weeks of topical treatment and five out of 11 patients with grade 2 eruption, who also received systemic minocycline 100 mg daily had an improvement in pustules and erythema of more than 50% [122]. Another prospective clinical study from 2009 by Scope A et al. which evaluated the ability of topical pimecrolimus to reduce the severity of cetuximab- associated facial eruption in patients with mCRC demonstrated that its application did not bring a meaningful clinical benefit for patients [123].

Vitamin K1-based cream may be another potential topical therapeutic option, even though data available is not conclusive. An Italian clinical trial from 2014 by Pinta F et al. showed that vitamin K1-based cream applied twice daily on the face and trunk from the first day of administration of cetuximab in mCRC patients led to a reduced proportion of grade 2 rash (22.5%) and also a lower proportion of grade 3 skin rash compared to values reported in literature [124]. Another controlled trial on 61 patients with mCRC evaluated the efficacy of topical vitamin K1 cream beginning on the first day of cetuximab treatment did not decrease the risk of acneiform rash [125]. In the PROSKIN study, physicians mentioned a moderate effectiveness of topical vitamin K1 cream concerning skin rashes induced by cetuximab treatment for CRC and squamous cell cancer of the head and neck patients [14].

Topical BRAF inhibitors were proposed as a promising option in the management of EGFR inhibitors-induced papulopustular rash while minimizing systemic adverse effects [126,127]. Skin toxicity appears to be due to the inhibition of mitogen-activated protein kinase (MAPK) signaling by EGFR inhibitors. Contrary, BRAF inhibitors have the ability to activate downstream the MAPK pathway [127]. In a phase I clinical trial from 2020, Lacouture ME et al. studied a new topical BRAF inhibitor, LUT014, in the management of the acneiform rash in 10 patients with metastatic CRC under EGFR inhibitors (panitumumab or cetuximab) [127]. Results showed that six patients with grade 2 rash had their acneiform eruption improved [127]. LUT014 did not exert dose-limiting toxicities and was well tolerated, thereby, the authors concluded that topical BRAF inhibitors presented safety and efficacy in improving skin toxicity in patients under EGFR inhibitors [127]. This is in accordance with another review from 2020 by Wang CJ and Brownell I on BRAF inhibitors for the therapeutic approach of papulopustular rashes from MAPK pathway inhibitors [126].

Recent publications suggest that systemic retinoids may represent possible alternatives to antibiotic treatment in patients with acneiform eruptions under EGFR inhibitors. In March 2021 was published a retrospective study by Costello CM et al. on six patients with EGFR inhibitor/small molecule tyrosine kinase inhibitor acneiform eruption of which four were treated with isotretinoin (10 mg to 60 mg daily), one with acitretin (10 mg to 25 mg daily) and one with isotretinoin and, subsequently, with acitretin. Results showed that isotretinoin led to a greater clinical improvement compared to acitretin with respect to acneiform eruptions [128]. These results are in accordance with those of Andrews ED et al. from 2020, who also reported that patients with acneiform eruption due to EGFR inhibitors taking acitretin or isotretinoin within one month maintained a stable Grade 1 dermatitis without dose reduction of oncologic therapy [129]. In a case series from 2020 by Caruana M et al., three patients treated with trametinib, a selective inhibitor of mitogen-activated protein kinase (MEK inhibitor), who developed an acneiform rash as the most frequent adverse reaction presented clinical cutaneous improvement in one to three months after low-dose isotretinoin (0.15 to 0.35 mg/kg/d) [130]. The retinoid therapy was well tolerated by all three patients, without significant changes in triglycerides levels or increased hepatotoxicity [130]. Routine laboratory testing with fasting lipid panel and liver function tests is recommended during isotretinoin therapy [130]. The use of retinoids in advanced colon cancer with liver metastasis may be worrisome for clinicians and needs more investigation.

While alternative therapies might seem appealing to spare long-term preemptive antibiotic therapy, there is insufficient data from randomized controlled trials to propose an efficient and safe algorithm. In the future, combined therapies targeting multiple pathogenic pathways may show promising results.

## 5. Limitations of the Current Knowledge

Due to the complexity of the interactions which are established between microbiota and human host and to the significant influence of numerous external factors (e.g., diet, antibiotic, other treatments), the subject of this review is requiring further studies to adequately confirm this hypothesis.

Metastatic CRC patients already have a pre-existing dysbiosis, with *Fusobacterium* species dominating the gut microenvironment [131]. Moreover, the composition of the intestinal microbiome is highly influenced by multiple factors. Ageing process may affect the human gut microbiota structure, even though a study found that young adults and elderly adults present similarities, with *Bacteroidetes* and *Firmicutes* being highly dominant [132]. Diet is another factor that influences gut microbiota, it has been shown that intake of fruits, vegetables and fibers is associated with a high amount of *Firmicutes* species [133]. A recent study revealed that food intake could explain up to 24% of human gut microbiome variation [134]. Antibiotics, as mentioned above, also influence the structure of gut microbiome, as well as probiotics, prebiotics (ingredients which contain non-digestible oligosaccharides) and synbiotics (associations of probiotics and prebiotics) [104,135].

In addition to the impact of long-term antibiotic therapy on gut microbiome in patients receiving EGFR inhibitors, modifications of skin microbiome due to infections that complicate dermatologic toxic effects associated with EGFR inhibitors may also represent a subject of interest for further research. A retrospective review from 2010 by Eilers Jr RE et al. on 221 cancer patients under EGFR inhibitors aimed to examine the prevalence of infections that complicate cutaneous toxicity of these targeted-therapies [136]. Results of bacterial cultures, immunohistochemical staining of skin specimens for viral pathogens and histopathologic assessment of biopsy samples were reviewed: 84 subjects (38%) showed evidence of infection at sites of dermatologic side effect; 50 (22.6%) of the patients had cultures positive for *Staphylococcus aureus* [136]. Less frequently, patients had infection with herpes simplex (3.2%), herpes zoster (1.8%) and dermatophytes (10.4%). The seborrheic region was the most prevalent site of infection [136]. This is in accordance with evidence from literature which shows that the acneiform rash is the most frequent dermatologic side effect of EGFR inhibitors, usually occurring in these particular anatomical sites [137]. Several other studies on the cutaneous adverse reactions in cancer patients under EGFR inhibitors were mentioned in depth throughout the article.

Another limitation is the reduced survival of mCRC patients. A population-based analysis from January 2020 by Wang J et al. highlighted that the three years OS was 20.7% and the five years OS was 10.5% in patients with stage IV colon adenocarcinoma [138]. The site of metastasis was a significant prognostic factor for OS and DSS and lung-only metastasis was associated with better OS an DSS values compared to liver-only metastasis [138]. Other independent predictors of poor OS were older age, high preoperative values of carcinoembryonic antigen (CEA), no surgery of the primary site, no chemotherapy and advanced tumor-node-metastasis (TNM), to name a few [138].

## 6. Conclusions

In this article, we reviewed studies which referred to the potential impact of long-term antibiotic therapy in patients with CRC. At present, there is insufficient data regarding the effects of antibiotic therapy for cutaneous side effects of EGFR inhibitors in CRC patients. Tetracyclines seem a viable therapeutic option, either preemptive (preferable) or reactive, usually well tolerated by oncologic patients. Future prospective and randomized studies are needed in order to provide evidence for the main issues addressed in this article: bacterial ‘fitness’ leading to antibiotic resistance and/or increased microbial virulence, gut dysbiosis in vulnerable CRC patients, adverse reactions related to tetracyclines, the impact on the response to oncologic therapy and disease prognosis. Moreover, studies should address the use of maximal doses vs. slow release tetracyclines with lower concentrations in CRC patients, with potential benefits of the second option.

The pre-existing dysbiosis remains a major concern in CRC patients. Modification of gut microbiome through either microbiota transplantation or administration of probiotics, prebiotics, postbiotics and synbiotics may represent an effective solution, as presented above. Future studies may be needed to demonstrate the safety and efficacy of this therapeutic approaches.

*Fusobacterium nucleatum* is the main bacterial species of the gut microbiome in CRC, playing a key role in tumorigenesis, resistance to chemotherapy and prognosis, as described anteriorly. Development of an antibiotic with targeted anti-*Fusobacterium* activity may, as well, represent a future field of research, since such therapeutic agent could be of a potential benefit for patients with CRC.

## Data Availability

No new data were created or analyzed in this study. Data sharing is not applicable to this article.
